# Complications of the Fingers and Hand After Arthroscopic Rotator Cuff Repair

**DOI:** 10.2174/1874325001812010134

**Published:** 2018-03-30

**Authors:** Mikio Harada, Nariyuki Mura, Masatoshi Takahara, Michiaki Takagi

**Affiliations:** 1 Department of Orthopedic Surgery, Izumi Orthopedic Hospital, Maruyama Aza Kamiyagari 6-1, Izumi-ku, Sendai, Miyagi, 981-3121, Japan; 2 Department of Orthopedic Surgery, Yoshioka Hospital, Higashihon-cho 3-5-21, Tendo, Yamagata, 994-0026, Japan; 3 Department of Orthopedic Surgery, Yamagata University Faculty of Medicine Iida-Nishi-2-2-2, Yamagata, 990-9585, Japan

**Keywords:** Rotator cuff, Complication, Cubital tunnel syndrome, Carpal tunnel syndrome, Flexor tenosynovitis, Complex regional pain syndrome, Finger

## Abstract

**Background:**

Complications of the fingers and hand that occur after Arthroscopic Rotator Cuff Repair (ARCR) have not been examined in detail.

**Objective:**

The aim of our study was to evaluate the diagnosis and treatment of complications of the fingers and hand that occur after ARCR and to examine treatment outcomes.

**Methods:**

The case records of 40 patients (41 shoulders) who underwent ARCR using suture anchors were retrospectively reviewed to investigate complications of the fingers and hand after ARCR.

**Results:**

Twelve patients (29%) experienced numbness, pain, edema, and movement limitations of the fingers and hand. These symptoms occurred on average 1.1 months (range, 0.1-2.5 months) after ARCR. The diagnoses were cubital tunnel syndrome in 2 hands, carpal tunnel syndrome in 3 hands, and flexor tenosynovitis (TS) in 10 hands. None of the 10 hands with TS exhibited triggering of the fingers. The mean interval between treatment initiation and symptom resolution was 2.2 months for the 5 hands treated by corticosteroid injection or surgery and 5.9 months for the 7 hands treated by alternating warm and cold baths alone. None of the hands exhibited Complex Regional Pain Syndrome (CRPS).

**Conclusion:**

Complications of the fingers and hand after ARCR were observed in 29%. TS was the most frequent complication. When symptoms in the fingers and hand occur after ARCR, rather than immediately suspecting CRPS, TS should be primarily suspected, including when TS symptoms such as triggering are not present, and these patients should be treated proactively using corticosteroid injections or surgery.

## 1 INTRODUCTION

Shoulder-hand syndrome is a reflex neurovascular disorder involving the shoulder and hand. It is characterized by a peculiar combination of painful shoulder disability and unilateral pain, swelling, and motion limitations of the hand and fingers [1-3]. While the pathophysiological mechanism of shoulder-hand syndrome has not yet been established, both trauma and surgical intervention may provoke reflex neurovascular reactions, which may result in the development of this syndrome [3]. Complex Regional Pain Syndrome (CRPS) type I is a serious complication of the fingers and hand that occurs after Arthroscopic Rotator Cuff Repair (ARCR) [4-13]. CRPS type I that results from trauma or surgical intervention, is a clinical entity characterized by severe spontaneous pain that is disproportionate to the inciting event and by motor symptoms, such as movement limitations of the fingers and hand. The prevalence of CRPS ranges from 0 to 12% after open rotator cuff repair [11, 14] and from 0 to 14.8% after ARCR [4, 8, 11, 15-17]. Additionally, the prevalence of paresthesia, including finger numbness on the operated side after ARCR, was reported to range from 10 to 30%, with most episodes of paresthesia reported as transient [7, 18-22]. However, the diagnosis and treatment of numbness, pain, edema, and movement limitations of the fingers and hand occurring early after ARCR have not been examined in detail. Moreover, various disorders with symptoms and signs similar to those of CRPS type I have been reported, and Carpal Tunnel Syndrome (CTS) and flexor tenosynovitis (TS) were shown to occur after trauma or surgical intervention, resulting in the presentation of CRPS-like symptoms [23, 24]. Notably, no study has assessed the prevalence of CTS or TS after ARCR. The aim of the present study was to evaluate the diagnosis and treatment of complications of the fingers and hand that occur after ARCR and to examine treatment outcomes.

### 2 PATIENTS AND METHODS

Institutional review board approval was acquired before initiation of this study, and informed consent was obtained from all the patients. From April 2011 to March 2012, 113 patients underwent arthroscopic repair of full-thickness rotator cuff tears. We excluded patients with isolated subscapularis tears, partial tears, and revision surgery. Among these 113 patients, the case records of 40 patients (41 shoulders) who were followed for more than one year after surgery were retrospectively reviewed. The patients’ ages ranged from 38 to 80 years (mean, 60.5 years), and they included 28 males and 12 females. Additionally, the operated sides consisted of the dominant shoulder in 29 patients, the non-dominant shoulder in 10, and both shoulders in one patient. Some of these results were reported in previous studies by Harada *et al*. [25] and Konta *et al*. [26].

All the arthroscopic rotator cuff surgeries were performed by one of the senior authors (N.M.) as either the primary or assistant surgeon. After undergoing general anesthesia, all the patients were placed in the beach chair position (T-MAX Beach Chair, Smith & Nephew, Andover, MA, USA). A Spider Arm Holder (Spider, Smith & Nephew, Andover, MA, USA) was used during surgery on 39 shoulders and not used on 2 shoulders. Acromioplasty and ARCR using suture anchors were performed on all the patients. Among the 41 shoulders included, primary repair was performed on 39, and a fascia lata patch graft was used on the other 2 shoulders. According to the Cofield classification, the tear size was small (less than 1 cm) in 14 (34%), medium (between 1 and 3 cm) in 21 (51%), large (from 3 to 5 cm) in 4 (10%), and massive (more than 5 cm) in 2 shoulders (5%).

After surgery, the patients were immobilized using an UltraSling (UltraSling, DONJOY, Ontario, CA) for a period of 4 to 6 weeks. On postoperative day 1, all the patients in this consecutive series began an organized exercise program including active range of motion (ROM) of the fingers and elbow under the supervision of a physical therapist. The patients began performing passive ROM shoulder exercises at 1 to 4 weeks (mean, 1.8 weeks) after surgery and active ROM exercises at 8 weeks after surgery. The mean observational period after surgery was 15.4 months (range, 12-35 months).

Until one month after surgery, the patients were examined almost daily because they had been hospitalized for one month. From one to three months after surgery, the patients were examined at one- to two-week intervals. From three months after surgery to the final follow-up, the patients were examined at one- to three-month intervals. We recorded numbness, pain, edema, and movement limitations of the fingers and hand on the operated side that occurred after ARCR, as well as the occurrence times of these symptoms. Additionally, we assessed the diagnosis and treatment of these symptoms and treatment outcomes. A diagnosis of Cubital Tunnel Syndrome (CuTS) was based on clinical findings such as finger numbness (little or ring finger) or medial elbow pain, along with an abnormal Tinel’s sign and elbow flexion test results, sensory change and muscle weakness in the ulnar nerve distribution, and a delay in ulnar nerve conduction velocity on the operated side compared with that on the non-operated side. Moreover, the diagnosis of CTS was based on clinical findings such as finger numbness (thumb, index, middle, or ring finger) or pinch disorder, and abnormal findings for Tinel’s sign, Phalen’s test, and carpal compression test results, atrophy and muscle weakness of the abductor pollicis brevis, abnormal Perfect O sign, and a delay in distal motor latency of the median nerve beyond 4.2 milliseconds. A diagnosis of TS was based on clinical findings such as motion pain or movement limitations of the fingers and abnormal findings regarding more than one of the following: triggering, tenderness around the metacarpophalangeal (MP) joints, and swelling of the flexor tendon. Swelling of the flexor tendon was considered present when an orthopedic clinician noted thickening of the flexor tendon distal or proximal to the A1 pulley while pulling the volar MP joint as the patient actively flexed the fingers.

## 3 RESULT

Twelve patients (29%) experienced numbness, pain, edema, and movement limitations of the fingers and hand on the operated side after ARCR. Finger numbness was present in 4 hands (10%), including the thumb in 3, index finger in 2, middle finger in 1, ring finger in 2, and little finger in 2 hands (all 4 hands demonstrated numbness in multiple fingers). Finger numbness occurred on average 0.8 months (range, 0.1-1.9 months) after ARCR. Movement limitations with pain and edema of the fingers and hand were observed in 9 hands (22%) on average 1.2 months (range, 0.3-2.5 months) after ARCR. Among the 12 patients with complications, the diagnoses based on these symptoms included CuTS for 2 hands, CTS for 3, and TS for 10 (Table **1**). None of the 10 hands with TS exhibited triggering of the fingers. The mean interval between the occurrence and diagnosis of CuTS, CTS, and TS was 2.1 months (range, 0-5.2 months), with mean intervals of 1.4, 3.8, and 1.7 months for CuTS, CTS, and TS, respectively. The interval between the occurrence and diagnosis of CTS was longer than that of CuTS or TS. The treatments administered to the 12 patients and their outcomes are shown in Table **1**. The treatments consisted of alternating warm and cold baths for 9 hands (CuTS: 1 hand; and TS: 9 hands), corticosteroid injections for 4 hands (CTS: 2 hands; and TS: 3 hands), and surgery for 2 hands (CuTS: 1 hand; and TS: 1 hand). The mean interval between treatment initiation and symptom resolution was 4.3 months after surgery (range, 0.6-9.7 months) for all 12 patients, with mean intervals of 5.9 months (range, 0.6-9.7 months) for the 7 hands treated with alternating warm and cold baths alone and 2.2 months (range, 0.7-7.6 months) for the 5 hands treated with corticosteroid injection or surgery. None of the hands exhibited CRPS.

## 4 DISCUSSION

In our study, 12 patients (29%) experienced numbness, pain, edema, and movement limitations of the fingers and hand within 3 months after ARCR, and the diagnoses according to these symptoms included CuTS for 2 hands, CTS for 3, and TS for 10 hands. These finger/hand complications are common. The potential mechanisms for these complications are as follows: 1) exacerbation of symptoms due to a disease that existed before surgery; 2) symptom occurrence due to subclinical disease that existed before surgery; and 3) acute occurrence of symptoms due to a new disease that developed after surgery. However, we could not determine which mechanism was applicable to these complications because we did not perform screening examinations for finger and hand diseases before surgery. Finger numbness occurred on average 0.8 months after ARCR, and movement limitations with pain and edema of the fingers and hand occurred on average 1.2 months after ARCR. Thus, these symptoms occurred shortly after ARCR. Because the patients were restrained at the shoulder to within 20 degrees of abduction for a period of 4 to 6 weeks after ARCR, their finger and hand use was extremely limited; consequently, many of them experienced edema of the fingers and hand. This finding suggests that the ARCR surgery led to edema of the upper extremity, which promoted the development of these common conditions of the fingers and hand. This is the first study to focus on complications of the fingers and hand that occur after ARCR.

Berjano *et al*. [27] reported the development of CuTS after arthroscopic shoulder surgery in 3 of the 172 patients; these patients’ symptoms were not serious and resolved 2 to 12 weeks after surgery. Furthermore, another report described the development of CuTS in 2 patients after arthroscopic shoulder surgery [28, 29]. In these previous studies, the authors speculated that CuTS developed after surgery because the elbow and forearm were covered by an elastic bandage during surgery, and traction was also applied to the upper extremity on the operated side. In our study, 2 hands with CuTS exhibited finger numbness at 0.2 and 1.9 months after ARCR. The elbow flexion position during immobilization after ARCR was speculated to exert increased pressure on the cubital tunnel, resulting in the development of CuTS. To avoid this development, screening examinations for CuTS should be performed before surgery, and careful attention should be paid to the degree of elbow flexion during immobilization.

CTS and TS were reported to occur after trauma or surgical intervention, resulting in the presentation of CRPS-like symptoms [23, 24]. Our study also demonstrated the development of CTS and TS with CRPS-like symptoms after ARCR. To prevent worsening of these symptoms, immediately after the patients experienced movement limitations with pain and edema of the fingers and hand, we began to treat the symptoms using alternating warm and cold baths. The mean interval between treatment initiation and symptom resolution was 5.9 months for the 7 hands treated with alternating warm and cold baths alone. Additionally, for the 4 hands treated with corticosteroid injection, the symptoms resolved within 1 month after injection. One hand with TS, in which finger pain recurred after corticosteroid injection, was treated with surgery. These results suggest that when numbness, pain, edema, and movement limitations of the fingers and hand occur after ARCR, rapid corticosteroid injection administration may lead to improvement of these symptoms, thereby effectively preventing the development of CRPS. Moreover, none of the 9 patients with movement limitations and pain and edema of the fingers and hand exhibited triggering of the fingers. When numbness, pain, edema, and movement limitations of the fingers and hand occur after ARCR, it is crucial to not initially suspect CRPS, which is an unknown disease, but instead to primarily suspect TS, including when TS symptoms such as triggering are not present; these patients should be treated proactively using corticosteroid injections or surgery.

Because they had no history of CuTS, CTS, and TS before surgery, we believed that these finger/hand complications represented new conditions; however, because this study was retrospective, prospective studies are required to examine whether these complications represented new syndromes that developed after surgery. Limitations of this study included its retrospective review as well as the difficulties detecting common subclinical diseases of the fingers and hand before surgery and of quantitatively assessing pain or movement limitations of the fingers and hand.

## CONCLUSION

Among 40 patients (41 shoulders) who underwent ARCR, complications of the fingers and hand occurred after ARCR in 12 (29%) patients. The diagnoses according to the symptoms included CuTS for 2 hands, CTS for 3, and TS for 10. No hands with CRPS were observed. Complications of the fingers and hand occurred on average 1.1 months after ARCR, and these symptoms resolved in all the patients on average 4.3 months after ARCR. When numbness, pain, edema, and movement limitations of the fingers and hand occur after ARCR, it is important to not initially suspect CRPS, which is an unknown disease, but rather to primarily suspect TS, including when TS symptoms such as triggering are not present; these patients should be treated proactively using corticosteroid injections or surgery.

## Figures and Tables

**Table 1:** Patient details: diagnoses and treatment outcomes for numbness, pain, edema, and movement limitations of the fingers and hand.

Finger numbness					Movement limitations with pain and edema of the fingers and hand		Diagnosis					
Patient	Age (yrs)	Sex	Presence	Occurrence time (M)	Presence	Occurrence time (M)	CuTS*	Interval (M)^##^	CTS**	Interval (M)^##^	TS***	Interval (M)^##^
1	56	M	Yes	0.2	Yes	0.3	Yes	1.7	No	-	Yes	0
2	64	M	Yes	1.9	No	-	Yes	1.1	Yes	1.1	No	-
3	61	M	Yes	0.1	No	-	No	-	Yes	5.2	No	-
4	48	F	Yes	0.9	No	-	No	-	Yes	5.1	Yes	5.1
5	55	F	No	-	Yes	1.4	No	-	No	-	Yes	0
6	52	M	No	-	Yes	2.5	No	-	No	-	Yes	0
7	60	M	No	-	Yes	1.4	No	-	No	-	Yes	0
8	80	M	No	-	Yes	1.3	No	-	No	-	Yes	0
9	55	M	No	-	Yes	0.4	No	-	No	-	Yes	0
10	69	F	No	-	Yes	0.6	No	-	No	-	Yes	0
11	60	M	No	-	Yes	1.7	No	-	No	-	Yes	0
12	60	M	No	-	Yes	1.0	No	-	No	-	Yes	0

*CuTS: Cubital tunnel syndrome. **CTS: Carpal tunnel syndrome. ***TS: Flexor tenosynovitis. Yes^W,F^: W: Wrist (CTS), F: Finger (TS). # Patient 6: The symptoms resolved 1 month after injection; however, finger pain recurred, and he subsequently underwent surgery.Interval between occurrence and diagnosis (M). ### Interval between treatment initiation and symptom resolution (M).

**Table:** 

Interval between treatment initiation and symptom resolution (M)							
Patient	Alternating warm and cold baths	Interval (M)^###^	Injection	Interval (M)^###^	Surgery	Interval (M)^###^	Observational period after surgery (M)
1	Yes	9	-	-	-	-	15
2	-	-	-	-	Yes^E^	1	35
3	-	-	Yes	0.7	-	-	13
4	-	-	Yes^W,F^	1	-	-	29
5	Yes	0.7	Yes	0.7	-	-	25
6	Yes	7.6	Yes	1^#^	Yes	1	18
7	Yes	0.6	-	-	-	-	18
8	Yes	9.7	-	-	-	-	18
9	Yes	4.6	-	-	-	-	18
10	Yes	6.4	-	-	-	-	13
11	Yes	8.3	-	-	-	-	18
12	Yes	3	-	-	-	-	12
